# A Self-Supervised Specific Emitter Identification Method Based on Contrastive Asymmetric Masked Learning

**DOI:** 10.3390/s25134023

**Published:** 2025-06-27

**Authors:** Dong Wang, Yonghui Huang, Tianshu Cui, Yan Zhu

**Affiliations:** 1 Key Laboratory of Electronics and Information Technology for Space Systems, National Space Science Center, Chinese Academy of Sciences, Beijing 100190, China; wangdong20@mails.ucas.ac.cn (D.W.); yonghui@nssc.ac.cn (Y.H.); 2 University of Chinese Academy of Sciences, Beijing 100049, China; 3 China Academy of Aerospace Science and Innovation, Beijing 100080, China; cuitshu@163.com

**Keywords:** specific emitter identification, wireless device security, self-supervised learning, asymmetric masked auto-encoder, contrastive learning

## Abstract

Specific emitter identification (SEI) is a core technology for wireless device security that plays a crucial role in protecting wireless communication systems from various security threats. However, current deep learning-based SEI methods heavily rely on large amounts of labeled data for supervised training, facing challenges in non-cooperative communication scenarios. To address these issues, this paper proposes a novel contrastive asymmetric masked learning-based SEI (CAML-SEI) method, effectively solving the problem of SEI under scarce labeled samples. The proposed method constructs an asymmetric auto-encoder architecture, comprising an encoder network based on channel squeeze-and-excitation residual blocks to capture radio frequency fingerprint (RFF) features embedded in signals, while employing a lightweight single-layer convolutional decoder for masked signal reconstruction. This design promotes the learning of fine-grained local feature representations. To further enhance feature discriminability, a learnable non-linear mapping is introduced to compress high-dimensional encoded features into a compact low-dimensional space, accompanied by a contrastive loss function that simultaneously achieves feature aggregation of positive samples and feature separation of negative samples. Finally, the network is jointly optimized by combining signal reconstruction and feature contrast tasks. Experiments conducted on real-world ADS-B and Wi-Fi datasets demonstrate that the proposed method effectively learns generalized RFF features, and the results show superior performance compared with other SEI methods.

## 1. Introduction

In the current era of highly developed digitalization, wireless communication technologies have been widely applied in various fields. From the interconnection of smart home devices in the civilian domain to the transmission of critical instructions in the military domain, the security of wireless communication is of utmost importance [1,2]. For example, during the flight of an aircraft, it needs to maintain close contact with the ground station through wireless communication and transmit key information such as position, speed, altitude, and flight attitude in real time. The accurate and secure transmission of this information is crucial for ensuring flight safety. In the traditional encryption authentication method, the security of the encryption algorithm depends on the confidentiality of the key. Once the key is leaked, the entire communication system will face huge risks. Moreover, the encryption and decryption processes are computationally intensive, which will severely affect the performance of some resource-constrained devices [3,4]. Specific emitter identification (SEI) is a non-cryptographic authentication method based on device physical characteristics [5]. Due to hardware characteristic differences, such as I/Q imbalance [6], power amplifier non-linearity [7], and frequency offset [8], each transmitter leaves unique and hard-to-forge radio frequency fingerprints (RFFs) in the signal. SEI technology has significant advantages compared with cryptography-based methods and has great application value in fields such as cognitive radio, spectrum monitoring, and electronic countermeasures [9,10,11].

Traditional methods based on feature engineering rely heavily on expert knowledge and human experience. They manually extract features such as signal amplitude, phase, and frequency [12,13,14] and design classifiers to identify different transmitters. With the continuous increase in the number of wireless devices, the selection of effective fingerprint features becomes increasingly difficult, making it hard to adapt to the rapidly changing electromagnetic environment.

In recent years, deep learning (DL) [15] has achieved remarkable success in various fields, such as computer vision [16] and natural language processing [17]. With its outstanding automatic feature extraction capabilities, deep learning can extract the subtle feature patterns hidden in radio frequency signals from large amounts of data. Therefore, the deep learning method was applied to SEI. Some methods use the original In-phase and Quadrature (IQ) data as the input of convolutional neural networks (CNNs) [18,19,20] and recurrent neural network (RNNs) [21]. Merchant et al. [22] utilized a CNN to extract features from IQ signals for identifying ZigBee devices. The experimental results demonstrated the method’s robustness across various signal-to-noise ratios (SNRs). Sankhe et al. [23] proposed the ORACLE model, composed of two convolutional layers and two fully connected layers, to extract and fuse features from I and Q signals. Yu et al. [24] introduced a robust method based on a multisampling convolutional neural network (MSCNN), employing multiple downsampling transformations for multiscale feature extraction. Al-Shawabka et al. [25] fed IQ signals into the RFF-LSTM model, which consists of three stacked Long Short-Term Memory (LSTM) layers and one fully connected layer. In [26], the authors proposed the ConvRNN model, which combines convolutional layers with RNN layers to simultaneously extract spatial and temporal features from IQ signals. A lightweight Transformer-based GLFormer method was presented in [27]. This method automatically extracts long-term dependencies from IQ signals. Wang et al. [28] proposed the complex-valued neural network (CVNN), comprising nine complex-valued convolutional layers and two fully connected layers, along with the model compression method. This approach aims to reduce model complexity while maintaining accuracy.

Other methods adopt bispectrum transform [29], time–frequency transform [30], Hilbert–Huang transform [31], constellation diagrams [32], etc., as the input to extract the features in the transform domain. Shen et al. [33] used the time–frequency spectrograms obtained through Short-Time Fourier Transform (STFT) as input to the Transformer model. To overcome the impact of wireless channels, Shen et al. [34] proposed the channel independent spectrogram and data augmentation method to enhance the system’s robustness against channel variations. In [35], the authors processed the transmitter signals through empirical mode decomposition (EMD) and Hilbert transform (HT) into graph tensors and applied graph neural networks (GNNs) for graph classification. Peng et al. [32] introduced an SEI method based on a differential constellation trace figure (DCTF). The CNN was designed to recognize the DCTF features of different devices. Subsequently, Peng et al. [8] proposed the heat constellation trace figure (HCTF) method. Lin et al. [36] proposed converting the IQ signal into a contour stellar image (CSI) with statistical significance and then using a CNN model for classification.

However, DL-based SEI methods are data-driven and rely heavily on a large amount of labeled samples. Labeling massive samples requires expensive manual overhead, as it requires professional knowledge to accurately label each transmitter signal to determine its corresponding transmitter category. In many practical non-cooperative electromagnetic environments, only unlabeled samples can be obtained, and it is difficult to acquire a sufficient number of labeled data to train deep neural networks (DNNs) adequately, which often leads to overfitting of the model.

To fully leverage all unlabeled data, self-supervised learning (SSL) has been proposed. Unlike traditional supervised learning, SSL does not rely on a large number of labeled samples. Instead, it designs pre-training tasks that enable the model to generate high-quality feature representations, thereby enhancing the performance of downstream tasks. Contrastive and generative methods are two key paradigms in SSL pre-training. In the framework of contrastive methods, feature representations are learned by constructing positive and negative samples. Specifically, for a given input sample, two augmented views are generated. These augmented views are regarded as positive sample pairs relative to the original input sample. While the augmented views may differ in form due to data augmentation, they share the same semantic content. In contrast, augmented views derived from different input samples are considered negative samples. The model aims to minimize the distance between positive sample pairs and maximize the distance between positive and negative samples, thereby learning discriminative feature representations. Wu et al. [37] devised positive sample pairs via random slicing for contrastive learning, employing the pre-trained model as an initialization in the subsequent fine-tuning stage. Meanwhile, Liu et al. [38] utilized BYOL [39] as the backbone for self-supervised contrastive learning, pre-training an RFF extractor and introducing adversarial augmentation to enhance the self-supervised learning process. Thereafter, they utilized a limited set of labeled samples for fine-tuning the extractor and classifier. Inspired by SimCLR [40], Liu et al. [41] converted signals into constellation trace figures and adopted image augmentation techniques from the computer vision domain to generate extensive positive and negative sample pairs for contrastive learning. In contrast to contrastive methods, which emphasize distinguishing between different sample pairs, generative methods focus more on learning how to reconstruct or predict partially observed data. By enabling the model to learn the reconstruction of input data from latent representations or the generation of new samples consistent with the real data distribution, these methods capture the intrinsic structures and features of the data. Xie et al. [42] utilized the Hilbert time–frequency spectrum as the input to an auto-encoder for pre-training, followed by fine-tuning the pre-trained auto-encoder using a small amount of labeled data. Huang et al. [43] proposed a pre-training method based on a symmetric masked auto-encoder (MAE), which predicted the masked segments of signals. To achieve better feature extraction performance and reduce the computational cost associated with symmetric decoders, Yao et al. [44] introduced an asymmetric masked auto-encoder (AMAE) for pre-training the RFF extractor. Li et al. [45] proposed a CML framework for few-shot SEI, which decouples masked reconstruction and contrastive learning into a dual-branch architecture comprising an encoder–decoder branch for signal reconstruction and a momentum encoder branch for instance discrimination.

Inspired by the aforementioned analysis, contrastive learning demonstrates remarkable advantages in feature extraction by learning discriminative feature representations through the construction of positive–negative sample pairs. Meanwhile, generative methods effectively capture latent distribution characteristics via data reconstruction. While contrastive methods excel in inter-sample discriminability, they overlook critical fine-grained local patterns, whereas generative methods capture signal structures but lack explicit mechanisms for inter-class separation. Considering this, we bridge the gap by combining masked reconstruction with contrastive learning, which reinforces device identity discrimination through feature aggregation and separation. This approach achieves representation learning from local details to global discriminability. Distinct from the dual-branch architecture in [45], the proposed method employs a single-branch asymmetric auto-encoder incorporating a lightweight single-layer convolutional decoder. This design significantly reduces parameter and computational costs while retaining effective signal reconstruction capabilities. Furthermore, deviating from conventional random masking techniques, we introduce a novel complementary masking data augmentation strategy. This approach generates complementary masked views of identical signals, compelling the model to learn robust feature representations by correlating information from non-overlapping signal segments. To mitigate limitations in generic feature extraction, we integrate channel squeeze-and-excitation residual blocks within the encoder. These blocks dynamically recalibrate feature responses to enhance focus on discriminative radio frequency fingerprint regions. Additionally, a lightweight feature projection head is integrated to compress features into a compact, discriminative latent space. We further establish dual optimization objectives. Unlike traditional methods reconstructing the entire signal, the reconstruction task specifically targets the complementary masked regions. Simultaneously, the contrastive task explicitly optimizes inter-device discrimination. This dual focus on local detail reconstruction and global separability effectively overcomes the limitations of single-paradigm approaches.

To this end, we propose a novel contrastive asymmetric masked learning-based SEI (CAML-SEI) method, aiming to integrate the synergistic advantages of contrastive and generative learning to build robust and discriminative RFF feature representations. Specifically, we first employ a complementary masking data augmentation technique to construct positive and negative sample pairs for RFF feature representation contrastive learning. Subsequently, we design channel squeeze-and-excitation residual blocks (CSERBlocks) to build an encoder architecture that maps sample pairs into the feature space. Additionally, a lightweight feature projection head is introduced to eliminate redundant information while enhancing feature discriminability. Finally, we jointly optimize the network by combining masked signal reconstruction loss and contrastive loss. The main contributions of this paper are summarized as follows:

- An effective CAML-SEI method is proposed for SSL-SEI. This method enhances the model’s ability to extract RFF features and effectively overcomes the limitation of limited labeled samples.
- A novel asymmetric auto-encoder architecture is designed to implement CAML-SEI, comprising a CSERBlock-based encoder, a lightweight decoder, and a lightweight feature projection head.
- The pre-trained encoder is fine-tuned using only a limited number of labeled samples, and a simple classifier containing only one fully connected layer is trained to classify the RFF features for SEI.
- The proposed CAML-SEI method is evaluated on real-world ADS-B and Wi-Fi datasets. Simulation results show that the recognition performance of the proposed method is superior to other comparison methods.

## 2. Signal Model and Problem Formulation

### 2.1. Signal Model

Under the assumption that only one transmitter is active at any given time and its signal can be captured individually without confusion with other transmitters’ signals, the received signal from this transmitter can be described as follows:

(1) R(t)=h(t)∗s(t)+w(t),

where h(t) represents the channel impulse response between the receiver and the transmitter, s(t) denotes the original signal transmitted by the transmitter, and w(t) represents additive white Gaussian noise (AWGN). The symbol * here denotes the convolution operation. When the continuous-time signal R(t) is sampled at a sampling frequency $f_{s}$, a discrete complex sequence, known as IQ data, is obtained. The *k*-th signal is expressed as follows:

(2) $$x_{k}=\left[\begin{array}{c} R_{i}\left(0\right),R_{i}\left(1\right),\ldots ,R_{i}\left(L-1\right)\\ R_{q}\left(0\right),R_{q}\left(1\right),\ldots ,R_{q}\left(L-1\right) \end{array}\right].$$

where the size of $x_{k}$ is 2×L, with *L* being the length of each sampled signal sequence. k∈{1,2,…,N} is the index of the *k*-th signal, and *N* is the total number of signals. These IQ signals are used as inputs to the SEI model to identify the transmitters.

### 2.2. SEI Problem

Given the transmitter signal dataset $D={\left\{\left(x_{i},y_{i}\right)\right\}}_{i=1}^{N}$, where each $x_{i}\in X$ represents a signal and $y_{i}=\left[y_{i}^{\left(1\right)},y_{i}^{\left(2\right)},\ldots ,y_{i}^{\left(C\right)}\right]$ is the one-hot encoded label vector. When the sample belongs to the *c*-th transmitter, the label vector satisfies $y_{i}=\left[0,\ldots ,\underset{\overset{︸}{c\text{-}\mathrm{th}\;\mathrm{position}}}{1},\ldots ,0\right]$, where *C* denotes the total number of transmitters. Here, X and Y represent the signal space and class label space, respectively. The goal of SEI is to construct a mapping function $f_{SEI}:X\to Y$ by learning the relationship between signals and transmitters. The optimization objective can be formulated as minimizing the following loss:

(3) $$L_{sup}=\sum_{i=1}^{N}\ell_{sup}\left(f_{SEI}\left(x_{i}\right),y_{i}\right)$$

where $L_{sup}$ denotes a supervised loss function that quantifies the discrepancy between the model prediction $f_{SEI}\left(x_{i}\right)$ and the ground-truth label $y_{i}$. By minimizing this loss function, the model parameters can be optimized to ensure the predictions align closely with the true labels. However, when the number of labeled samples *N* is small (e.g., N=5 or 10), the model’s generalization capability deteriorates significantly, often leading to overfitting.

### 2.3. SSL-SEI Problem

In the SSL-SEI problem, the training dataset $D_{tr}$ consists of an unlabeled dataset $D_{ul}={\left\{x_{i}\right\}}_{i=1}^{N_{ul}}$ and a small labeled dataset $D_{l}={\left\{\left(x_{i},y_{i}\right)\right\}}_{i=1}^{N_{l}}$, i.e., $D_{tr}=D_{ul}\cup D_{l}$. Here, $N_{ul}$ denotes the number of unlabeled samples, $N_{l}$ denotes the number of labeled samples, and $N_{l}\ll N_{ul}$. During the pre-training stage, the feature encoder $f_{enc}$ is trained through the self-supervised learning task to learn general feature representations of the signals. The optimization objective at this stage can be formulated as minimizing the following loss:

(4) $$L_{pt}=\sum_{i=1}^{N_{ul}}\ell_{pt}\left(f_{enc}\left(x_{i}\right),T\right)$$

where $L_{pt}$ denotes the loss function of the self-supervised learning task and T is the target of the task. Upon completion of pre-training, the optimized parameters of the feature encoder $f_{enc}$ are saved. Subsequently, during the fine-tuning stage, the pre-trained feature encoder $f_{enc}$ is utilized: its learned parameters are loaded and serve as the initialization for the encoder within the SEI model. The entire SEI model (now comprising the pre-trained encoder $f_{enc}$ and a newly added classifier $f_{cls}$) is then further optimized using the labeled dataset $D_{l}$ to adapt to the SEI task. The loss function at this stage can be expressed as follows:

(5) $$L_{ft}=\sum_{i=1}^{N_{l}}\ell_{ft}\left(f_{SEI}\left(x_{i}\right),y_{i}\right)$$

where $L_{ft}$ represents the loss function of the supervised learning task, typically the cross-entropy loss, and $f_{SEI}=f_{enc}\circ f_{cls}$ denotes the combined SEI mapping function. Through this process of initializing the encoder $f_{enc}$ with pre-trained weights, the generic feature representations learned by the encoder during pre-training are effectively transferred to the fine-tuning stage. During fine-tuning, all model parameters (both $f_{enc}$ and $f_{cls}$) are further optimized to minimize $L_{ft}$. Ultimately, the model combining pre-training and fine-tuning achieves superior performance even with limited labeled data.

## 3. Proposed Method

**Figure 1:**
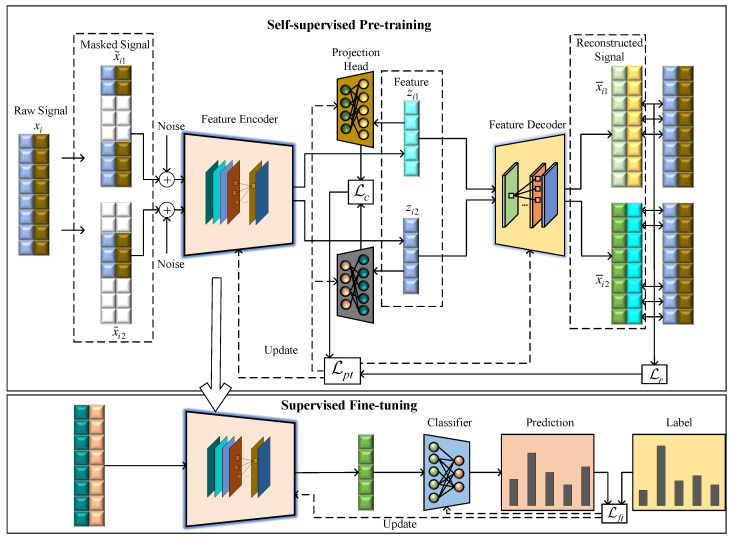
Contrastive asymmetric masked learning-based specific emitter identification method.

The proposed CAML-SEI method is illustrated in Figure 1. The framework incorporates a masked signal reconstruction task to enable the model to learn local structural characteristics of signals, while simultaneously employing a contrastive learning task to enhance intra-class compactness and inter-class separability among samples from similar devices. The joint optimization of these two objectives enables the model to simultaneously capture fine-grained features and establish globally discriminative representations. Furthermore, the CSERBlock is specifically designed to construct the feature encoder.

During the pre-training stage, only unlabeled samples are required to learn generalized signal characteristics. During the fine-tuning stage, the encoder parameters obtained from self-supervised pre-training are used as the initialization parameters for the encoder. The encoder and classifier undergo end-to-end training, ensuring the encoder can both learn generalized RFF features from unlabeled data and adapt to task specificity through a small number of labeled samples. Extensive experiments are conducted to validate the enhanced feature learning capability of the proposed method, which demonstrates advanced recognition performance compared with other methods.

### 3.1. Data Augmentation

Traditional masked signal reconstruction methods typically employ a single random mask for training by applying it to the input signal, while contrastive learning paradigms rely on similarity learning between paired augmented samples. The data augmentation in this paper consists of two steps, which are masking and adding Gaussian white noise. To achieve synergistic optimization of these two tasks, a complementary mask enhancement strategy is proposed to enable the model to infer complete signal features from different local information, thereby enhancing the comprehensiveness of feature extraction. The strategy generates two augmented samples with complementary mask positions from the original signal $x_{i}$ based on a preset masking ratio γ. Specifically, the starting point $m_{s}$ of the masked interval is randomly selected within the signal length *L*, and the ending point $m_{e}$ is calculated according to γ, ensuring that two mask blocks $m_{i1}$ and $m_{i2}$ operate on non-overlapping regions of the signal. $m_{i1}$ sets the interval $\left[m_{s},m_{e}\right]$ to zero while preserving the remaining parts, whereas $m_{i2}$ retains this interval and masks out other regions. This complementary masking facilitates the model in extracting effective features from distinct local segments and establishing cross-region feature associations, thereby effectively mitigating the potential issue of local feature overfitting that may arise from single-mask training. Additionally, after performing complementary masking, Gaussian white noise $v_{1}$ and $v_{2}$ are, respectively, added to the two masked signals to generate augmented samples ${\tilde{x}}_{i1}$ and ${\tilde{x}}_{i2}$:

(6) $$\gamma =\frac{m_{e}-m_{s}}{L}$$

(7) $${\tilde{x}}_{i1}=m_{i1}⊙x_{i}+v_{1}$$

(8) $$m_{i1}\left[n\right]=0,\;m_{s}⩽n⩽m_{e}$$

(9) $$m_{i1}\left[n\right]=1,\;0⩽n<m_{s},\;m_{e}<n<L$$

(10) $${\tilde{x}}_{i2}=m_{i2}⊙x_{i}+v_{2}$$

(11) $$m_{i2}\left[n\right]=1,\;m_{s}⩽n⩽m_{e}$$

(12) $$m_{i2}\left[n\right]=0,\;0⩽n<m_{s},\;m_{e}<n<L$$

where ⊙ denotes the Hadamard product. Therefore, the two complementarily masked signals obtained through the aforementioned data augmentation will serve as inputs to the feature encoder.

### 3.2. Network Structure

**Figure 2:**
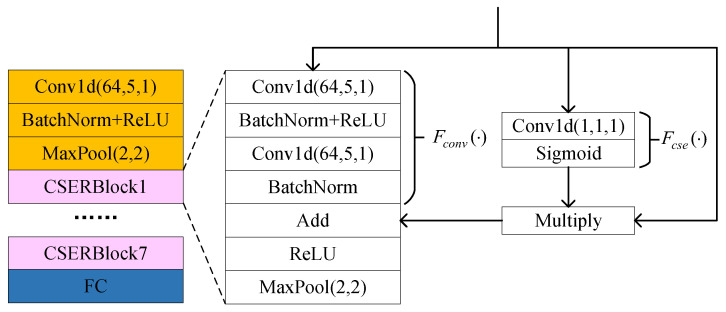
Structure of CSERNet.

To enhance the feature extraction capability of the encoder, we design an encoder architecture based on the CSERBlock, as illustrated in Figure 2. The encoder comprises a 1D convolutional layer, seven CSERBlocks, and a fully connected layer. Here, Conv1d (C, K, S) denotes a 1D convolutional layer with output channels C, kernel size K, and stride S. Here, the output channel number C is set to 64 to balance feature richness and computational efficiency. An insufficient number of channels would limit the model’s ability to capture key features, while excessive channels would increase computational overhead and easily lead to overfitting in few-shot scenarios. The convolution kernel size K is set to 5 as a trade-off choice to accommodate both short-term and long-term feature extraction. The stride S = 1 maximizes the retention of fingerprint details. These structural hyperparameters remain fixed throughout the training process, with only the convolution kernel weights being adaptively updated through backpropagation. In the CSERBlock, the input features are first transformed into a more compact representation through 1D convolution. In the “excitation” step, weights are generated via activation functions to adaptively recalibrate feature responses at different positions on the feature maps. This mechanism enables the model to focus on more informative regions, thereby optimizing the quality of feature representations. Simultaneously, residual connections ensure direct gradient flow, mitigating the vanishing gradient problem during the training of deep networks. Consequently, the residual block with channel squeeze-and-excitation enhances critical features while suppressing less significant ones by reweighting the importance of features at each position, thereby improving the model’s feature extraction capability. Finally, a max-pooling layer is employed to complete the feature extraction process.

Specifically, in the CSERBlock, the channel squeeze-and-excitation function $F_{cse}\left(\cdot \right)$ is composed of a 1D convolutional layer followed by the sigmoid activation function $\delta \left(\cdot \right)$ that maps inputs to values within the range of 0 to 1. By applying $F_{cse}\left(\cdot \right)$ to the input feature map $U\in R^{C\times L}$, the output response $r\in R^{1\times L}$ is generated as follows:

(13) $$r=F_{cse}\left(U\right)=\delta \left(conv1d\left(U\right)\right)$$

Each element $r_{i}$ in r indicates the relative importance of the feature at position *i*. The input feature map $U\in R^{C\times L}$ is then adjusted through the weighting mechanism, thereby recalibrating the importance of features at each position. This process enhances the influence of critical features while suppressing non-critical ones as follows:

(14) $$\tilde{U}=U\cdot r$$

where · denotes element-wise multiplication across channels. The excitation mechanism enhances focus on discriminative regions through a spatial attention mechanism that dynamically assigns location-specific weights to feature maps. Specifically, as defined in (13), a 1D convolutional layer compresses multi-channel features at each temporal position into a scalar value, followed by a sigmoid activation to generate a spatial weight vector. These weights recalibrate the original features via element-wise multiplication, where higher weights intensify responses to discriminative features, while lower weights suppress less informative regions. This weighting mechanism is adaptively optimized through backpropagation during training.

The function $F_{conv}\left(\cdot \right)$ consists of two 1D convolutional layers applied to the input feature map U. Its output is added to the recalibrated feature map $\tilde{U}$, thereby forming a residual connection. This residual connection ensures direct gradient flow, which helps mitigate the vanishing gradient problem during the training of deep networks. Finally, a max-pooling layer is utilized to achieve dimensionality reduction and feature extraction as follows:

(15) $${\tilde{U}}^{\prime }=\text{Maxpool}\left(\text{Re FU}\left(F_{conv}\left(U\right)+\tilde{U}\right)\right)$$

We adopt an asymmetric encoder–decoder architecture, where the decoder employs only a single 1D convolutional layer to reconstruct the signal. Compared to traditional symmetric encoder–decoder architectures, this asymmetric design significantly simplifies the decoder’s complexity. Due to the reduced parameters, the asymmetric decoder exhibits stronger generalization capability on training data, effectively mitigating the risk of overfitting. Furthermore, this design enhances flexibility and reduces demands on computational resources, thereby significantly lowering the model’s computational burden while maintaining efficient performance.

In contrastive learning tasks, two complementary masked augmented signals from the same signal may exhibit potential distribution shifts in their encoded features due to differences in semantic information density within masked regions. This asymmetric semantic distribution hinders the contrastive loss function from effectively minimizing the intra-class distance of positive sample pairs, thereby limiting the model’s capability to learn useful representations. Consequently, direct contrastive learning on raw features may encounter significant challenges in efficiently extracting discriminative feature representations. To address this issue, we introduce two lightweight feature projection heads subsequent to the feature encoder. Each projection head comprises merely a single fully connected layer, which employs non-linear projection to compress high-dimensional features into a low-dimensional space, thereby eliminating redundant information while enhancing feature discriminability. This design aims to mitigate distributional discrepancies among contrastive features and facilitate model optimization.

The classifier in the fine-tuning stage comprises a fully connected layer and a dropout layer to mitigate overfitting effects. Detailed network configurations are presented in Table 1.

### 3.3. Pre-Training Stage

During the self-supervised pre-training stage, two augmented masked signals ${\tilde{x}}_{i1}$ and ${\tilde{x}}_{i2}$ are fed into the feature encoder to extract latent fingerprint representations $z_{i1}=f_{enc}\left({\tilde{x}}_{i1}\right)$ and $z_{i2}=f_{enc}\left({\tilde{x}}_{i2}\right)$. For the signal reconstruction task, the latent representations are passed to the decoder to produce reconstructed signals ${\bar{x}}_{i1}=f_{dec}\left(z_{i1}\right)$ and ${\bar{x}}_{i2}=f_{dec}\left(z_{i2}\right)$. To minimize the discrepancy between reconstructed and original signals, the mean squared error (MSE) is employed as the loss function, with the loss calculated exclusively on the masked regions between the decoder predictions and the original signals:

(16) $$L_{r}=\frac{1}{2B}\sum_{i=1}^{B}\left({∥\left(x_{i}-{\bar{x}}_{i1}\right)⊙\left(1-m_{i1}\right)∥}^{2}+{∥\left(x_{i}-{\bar{x}}_{i2}\right)⊙\left(1-m_{i2}\right)∥}^{2}\right)$$

where *B* denotes the training batch size. Since $m_{i1}\cap m_{i2}=\emptyset$, the model must fully reconstruct the signal from these two mutually exclusive subsets. This compels the encoder to learn a global feature representation from local fragments and maintain semantic consistency across fragments. For random masking, due to the existence of overlapping regions, the model might neglect feature extraction from certain regions. For the latent fingerprint features $z_{i1}$ and $z_{i2}$ of two augmented samples, the features are further compressed via projection heads to obtain $z_{i1}^{\prime }=f_{pro1}\left(z_{i1}\right)$ and $z_{i2}^{\prime }=f_{pro2}\left(z_{i2}\right)$. We utilize cosine similarity as the similarity metric. Its advantage lies in its robustness to the magnitudes of feature vectors, enabling the model to focus on learning discriminative semantic features. The cosine similarity between them is computed as follows:

(17) $$\mathrm{Cos}\left({z_{i1}}^{\prime },{z_{i2}}^{\prime }\right)=\frac{\langle {z_{i1}}^{\prime },{z_{i2}}^{\prime }\rangle }{∥{z_{i1}}^{\prime }∥\cdot ∥{z_{i2}}^{\prime }∥}$$

To establish a discriminative feature representation space, we further design a contrastive learning-based optimization objective. Specifically, for positive signal pairs (i.e., the two masked augmented signals) derived from the same original signal, their latent features are encouraged to be close to each other, while being repelled from negative signal features originating from different signals. Formally, given a training batch containing *B* signals, for each signal $x_{i}$, the features of its two augmented versions, ${z_{i1}}^{\prime }$ and ${z_{i2}}^{\prime }$, form a positive pair. The negative signal set comprises the complementary mask signal features from other signals within the batch. Based on this construction, the contrastive loss is formulated as follows:

(18) $$L_{ci1}=-\log\frac{\exp(\mathrm{Cos}\left({z_{i1}}^{\prime },{z_{i2}}^{\prime }\right))}{\exp\left(\mathrm{Cos}\left({z_{i1}}^{\prime },{z_{i2}}^{\prime }\right)\right)+\sum_{j=1,j\neq i}^{B}\exp(\mathrm{Cos}\left({z_{i1}}^{\prime },{z_{j2}}^{\prime }\right))}$$

Similarly, $L_{ci2}$ is defined as

(19) $$L_{ci2}=-\log\frac{\exp(\mathrm{Cos}\left({z_{i2}}^{\prime },{z_{i1}}^{\prime }\right))}{\exp\left(\mathrm{Cos}\left({z_{i2}}^{\prime },{z_{i1}}^{\prime }\right)\right)+\sum_{i=1,j\neq i}^{B}\exp(\mathrm{Cos}\left({z_{i2}}^{\prime },{z_{j1}}^{\prime }\right))}$$

The total contrastive loss is defined as

(20) $$L_{c}=\frac{1}{2B}\sum_{i=1}^{B}\left(L_{ci1}+L_{ci2}\right)$$

In this way, the model learns to discriminate between positive signals derived from the same signal and negative signals derived from different signals, thereby improving the discriminative capability of latent features. The overall learning objective is formulated as a combination of the reconstruction loss $L_{r}$ and the contrastive loss $L_{c}$, defined as

(21) $$L_{pt}=L_{r}+L_{c}$$

Since the model is required to recover complete signals from masked inputs, the reconstruction loss facilitates the model in capturing the inherent structures and patterns of the signals, thereby enabling the learning of more comprehensive and enriched feature representations. The contrastive loss focuses on optimizing both the matching degree between two augmented signals and the contrastiveness between signals. This mechanism encourages the learned features to exhibit desirable instance discriminability.

### 3.4. Fine-Tuning Stage

After completing the parameter optimization of the feature encoder during the pre-training stage, we propose to transfer the pre-trained weights of the encoder as initial parameters to the model fine-tuning stage. This design effectively leverages the general feature representation capabilities acquired through self-supervised pre-training, thereby providing a more efficient initial foundation for subsequent fine-tuning tasks. Additionally, the classifier incorporates a fully connected layer. To minimize the discrepancy between the probability distribution ${\hat{y}}_{i}=\left[p_{i}^{\left(1\right)},p_{i}^{\left(2\right)},\ldots ,p_{i}^{\left(C\right)}\right]$ predicted by the model and that of the ground-truth labels, the cross-entropy loss function is employed as the optimization objective:

(22) $$L_{ft}=-\frac{1}{N_{lb}}\sum_{i=1}^{N_{lb}}\sum_{c=1}^{C}y_{i}^{\left(c\right)}\log p_{i}^{\left(c\right)}$$

where $N_{lb}$ is the batch size for model training in the fine-tuning stage. The training procedure of CAML-SEI is summarized in Algorithm 1.

**Algorithm 1** Training procedure of CAML-SEI method.
**Input:**

$D_{ul}={\left\{x_{i}\right\}}_{i=1}^{N_{ul}}$, $D_{l}={\left\{\left(x_{i},y_{i}\right)\right\}}_{i=1}^{N_{l}}$: Unlabeled and labeled datasets, respectively;$N_{pt}$, $N_{ft}$: Number of pre-training and fine-tuning epochs, respectively;$T_{pt}$, $T_{ft}$: Number of pre-training and fine-tuning iterations, respectively;$W_{enc}$, $W_{dec}$, $W_{cls}$: Parameters of the feature encoder, feature decoder, and classifier, respectively;$W_{pro1}$, $W_{pro2}$: Parameters of projection heads;η: Learning rate.
**Output:** Trained parameters $W_{enc}$ and $W_{cls}$.  **Pre-training Stage:**
1:**for** $k=1\;\mathrm{to}\;N_{pt}$ **do**2:  **for** $t=1\;\mathrm{to}\;T_{pt}$ **do**3:   Sample a batch of training signals from $D_{ul}$;4:   Generate two batches of masked signals ${\tilde{x}}_{1}$ and ${\tilde{x}}_{2}$ via data augmentation;5:   Feed masked signals into the feature encoder: $z_{1}=f_{enc}\left({\tilde{x}}_{1};W_{enc}\right)$, $z_{2}=$6:   $f_{enc}\left({\tilde{x}}_{2};W_{enc}\right)$;7:   Project latent features via heads: $z_{1}^{\prime }=f_{pro1}\left(z_{1};W_{pro1}\right)$, $z_{2}^{\prime }=f_{pro2}\left(z_{2};W_{pro2}\right)$;8:   Reconstruct signals using the decoder: ${\bar{x}}_{1}=f_{dec}\left(z_{1};W_{dec}\right)$, ${\bar{x}}_{2}=f_{dec}\left(z_{2};W_{dec}\right)$;9:   Compute reconstruction loss $L_{r}$ via Equation (16);10:   Compute contrastive loss $L_{c}$ via Equations (18)–(20);11:   Total loss: $L_{pt}=L_{r}+L_{c}$;12:   Update encoder: $W_{enc}\leftarrow W_{enc}-\eta \nabla_{W_{enc}}L_{pt}$;13:   Update projection heads: $W_{pro1}\leftarrow W_{pro1}-\eta \nabla_{W_{pro1}}L_{pt}$; $W_{pro2}\leftarrow W_{pro2}-$14:   $\eta \nabla_{W_{pro2}}L_{pt}$;15:   Update decoder: $W_{dec}\leftarrow W_{dec}-\eta \nabla_{W_{dec}}L_{pt}$;16:  **end for**17:
**end for**
18:Save feature encoder parameters;
  **Fine-tuning Stage:**
19:Load pre-trained encoder parameters;20:**for** $k=1\;\mathrm{to}\;N_{ft}$ **do**21:  **for** $t=1\;\mathrm{to}\;T_{ft}$ **do**22:   Sample a batch from $D_{l}$;23:   Extract features: $z=f_{enc}\left(x;W_{enc}\right)$;24:   Predict labels: $\hat{y}=f_{cls}\left(z;W_{cls}\right)$;25:   Compute cross-entropy loss $L_{ft}$ via Equation (22);26:   Update encoder: $W_{enc}\leftarrow W_{enc}-\eta \nabla_{W_{enc}}L_{ft}$;27:   Update classifier: $W_{cls}\leftarrow W_{cls}-\eta \nabla_{W_{cls}}L_{ft}$;28:  **end for**29:
**end for**



## 4. Experimental Results and Discussions

### 4.1. Dataset and Experimental Setup

The model performance is evaluated using a real-world collected ADS-B dataset containing 30 emitters and an open-source Wi-Fi dataset [23] containing 16 emitters. The ADS-B dataset is constructed based on a receiver system comprising the AD9361 (Analog Devices, Norwood, MA, USA) RF front-end and the ZYNQ-7020 (Xilinx, San Jose, CA, USA) programmable logic platform, with the system’s center frequency set to 1090 MHz and a sampling rate of 40 MHz. The SNR of ADS-B signals is 12–15 dB. Each raw signal frame contains 4780 sampling points, which are segmented into samples using a sliding window with a length of L=4000 and a step size of S=195. During the pre-training stage, 100 signal frames per aircraft are selected and sliced to generate 500 training samples; in the testing stage, 80 signal frames per aircraft are sliced to produce 400 test samples. The Wi-Fi dataset consists of signals complying with the IEEE 802.11a standard [46] and has the SNR of 30 dB. It is collected using a USRP B210 (Ettus Research, Austin, TX, USA) device, with a center frequency of 2450 MHz. The signal sources are 16 USRP X310 (Ettus Research, Austin, TX, USA) devices. The signals are generated using a sliding window with a fixed window length of L=4000 and a stride of S=4000. Each device contains 1000 signals in the pre-training stage and 800 signals per device in the testing stage. In the fine-tuning stage, experiments are conducted with 10, 15, 20, and 25 labeled signals per emitter.

During the pre-training stage, the batch size is set to 128, and the number of training epochs is set to 100. The Adam optimizer is employed with a learning rate of 0.001. The masking ratio γ is set to 0.4. The random seed is 300. In the fine-tuning stage, the same batch size of 128 is maintained, with the number of training epochs set to 200. The Adam optimizer is used with a learning rate of 0.001, coupled with a CosineAnnealingLR scheduler for learning rate adjustment. All our experiments were executed on a NVIDIA GeForce RTX 3090 GPU.

### 4.2. Comparison with Baseline Methods

The proposed method is compared with various generative approaches, including AE [47], DCGAN [48], MAE [43], AMAE [44], as well as contrastive learning methods, such as BYOL [39], SimCLR [40], and SA2SEI [38]. We conducted ten independent experiments and calculated statistical indicators including mean accuracy and standard deviation. Experimental results demonstrate that the proposed method exhibits significant performance advantages in two SEI tasks. Table 2 presents the performance comparison of various methods on the ADS-B dataset with 10, 15, 20, or 25 labeled samples per class. The results show that the proposed method significantly outperforms other methods and demonstrates robustness to label scarcity. The core robustness of our approach lies in its stable performance under extremely limited labeled data scenarios. Specifically, with only 10 labeled samples per class, the proposed method achieves a recognition accuracy of 78.29%, surpassing the suboptimal baseline method AMAE (74.04%) by 4.25%. As the number of labeled samples increases to 25 per class, the accuracy of the proposed method further improves to 88.00%, representing a 2.74% enhancement over the suboptimal AMAE approach. This indicates that the robust feature representation constructed during the self-supervised pre-training stage effectively captures the fingerprint characteristics of ADS-B signals.

Table 3 demonstrates the performance of different methods on the Wi-Fi dataset. Compared to baseline methods, the proposed CAML-SEI method consistently outperforms in four few-shot labeled sample scenarios. When the number of labeled samples is 10, CAML-SEI achieves an accuracy of 96.37%, outperforming the suboptimal baseline method MAE by 4.23%. As the number of labeled samples increases to 25, the accuracy of CAML-SEI further improves to 99.53%. It is noteworthy that the contrastive learning method SimCLR achieves an accuracy of 99.04% with 25 labeled samples per class, which is close to the proposed method’s accuracy of 99.53%. However, when only 10 labeled samples per class are available, SimCLR’s accuracy drops to 85.92%, which is 10.45% lower than the 96.37% achieved by the proposed CAML-SEI method. These results fully validate the robustness and effectiveness of the proposed method under limited labeled data conditions. Under the challenging condition of only 10 to 25 samples per class, the sustained lead fully demonstrates its strong tolerance to label scarcity, making it a reliable solution for few-shot SEI tasks.

**Figure 3:**
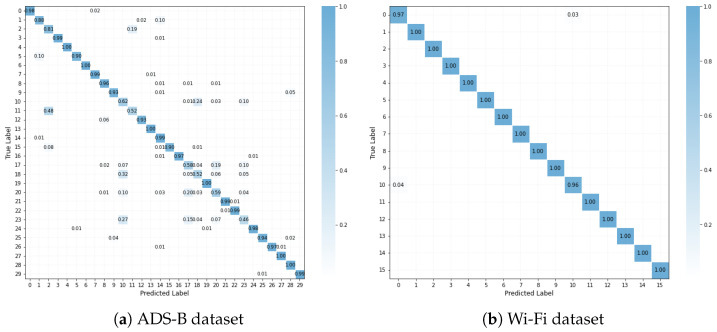
Confusion matrices.

**Figure 4:**
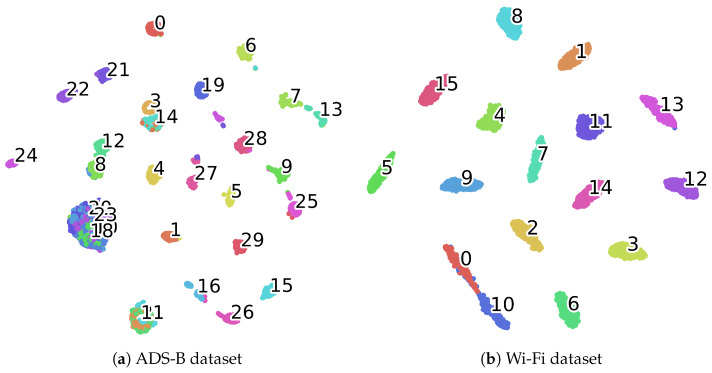
Feature visualization using t-SNE. Each color represents the feature distribution of the device signal, with numbers indicating the corresponding devices.

Table 4 and Table 5 present the F1-score and recall of the proposed CAML-SEI under different numbers of labeled samples. It can be observed that the F1-score and recall are generally consistent with the accuracy, which indicates that the model has relatively balanced prediction performance across all categories and shows certain robustness in terms of class balance. Figure 3 presents the confusion matrices of the proposed method on two datasets. The vast majority of the diagonal elements have very high values, intuitively confirming that most transmitters can be correctly identified. The t-SNE method is adopted to visualize the features learned by the model. As illustrated in Figure 4, the visualization results on two datasets demonstrate that the features of most categories form distinct clusters in the low-dimensional embedded space, exhibiting intra-class compactness and inter-class separation characteristics. This high degree of intra-class compactness and inter-class separation is a direct manifestation of the feature discriminability successfully achieved by the mechanism of the contrastive loss $L_{c}$. Through its loss function design, $L_{c}$ effectively pulls together the different views of the same signal while pushing apart views from different signals. Although some classes in Figure 4a may overlap partially due to environmental factors such as noise and different flight states, the majority of classes still form clear clusters, which confirms the strong discriminability of CAML-SEI for global features.

### 4.3. Ablation Study

Table 6 and Table 7 present the experimental results on two datasets, where “w/o CSE” denotes removing the channel squeeze-and-excitation operation, “w/o Proh” indicates removing the feature projection head, “w/o $L_{r}$” represents eliminating the reconstruction loss, and “w/o $L_{c}$” signifies the removal of the contrastive loss. “only $L_{c}$” means using only $L_{c}$, while removing the components CSE, Proh, and $L_{r}$. “only $L_{r}$” means using only $L_{r}$, while removing the components CSE, Proh, and $L_{c}$. The results demonstrate that all components contribute significantly to model performance improvement across a different number of labeled samples scenarios.

The ablation of the CSE module exhibits a particularly notable impact on model performance. On the ADS-B dataset with 10 labeled samples per class, the recognition accuracy drops by 10.99%, while a 4.96% decrease is observed on the Wi-Fi dataset under the same conditions. These results strongly validate the critical role of the feature weighting mechanism in CSERBlock. RFF features are generated by hardware defects and exhibit fine-grained local patterns in signals. The CSE module dynamically enhances responses in key feature regions by reweighting features. When the CSE module is removed, discriminative regions become averaged out, which causes the model’s ability to identify genuine RFF features to diminish, thus leading to a decline in recognition performance. The removal of the feature projection head negatively affects both datasets, causing accuracy reductions of 1.61% on the ADS-B dataset and 0.76% on the Wi-Fi dataset when 15 samples per class are labeled. Further analysis of loss function impacts reveals that the ablation of reconstruction loss leads to significant performance degradation on both datasets. With 10 labeled samples per class, the accuracy decreases by 5.64% on the ADS-B dataset and 3.75% on the Wi-Fi dataset, confirming the necessity of masked reconstruction for fine-grained feature extraction. This significant performance decline clearly demonstrates that the reconstruction task is crucial for the model to learn the inherent structures and patterns of signals. Removing it prevents the model from effectively learning comprehensive and enriched feature representations from masked signals, thereby impairing downstream classification performance. The reconstruction task forces the model to recover masked signals from complementary masked samples, enabling the capture of structural detail features in signals. When the reconstruction loss is removed, the model neglects these local features and tends to learn global features. However, such features are prone to lacking device specificity, which ultimately results in a drop in recognition performance. The removal of the contrastive loss has an even more pronounced impact, causing a 37.12% accuracy drop on the Wi-Fi dataset and a 14.09% drop on the ADS-B dataset when there are 10 labeled samples per class. This proves the core role of contrastive loss in optimizing the matching degree of positive sample pairs and the contrastiveness between positive and negative samples. Without $L_{c}$, the features learned by the model severely lack the ability to distinguish between different signal instances, leading to a substantial deterioration in classification performance. The design of contrastive loss explicitly achieves this goal by maximizing the similarity ratio between positive sample pairs versus negative sample pairs. Additionally, using either the reconstruction loss or the contrastive loss alone leads to a decrease in recognition accuracy. For example, on the ADS-B dataset with 10 labeled samples per class, using only the reconstruction loss results in a 15.74% drop in accuracy; on the Wi-Fi dataset, it leads to a 30.72% drop. On the ADS-B dataset with 10 labeled samples per class, using only the contrastive loss results in a 5.68% drop in accuracy; on the Wi-Fi dataset, it leads to a 6.13% drop. The complete model that uses both $L_{r}$ and $L_{c}$ consistently outperforms the models using either loss function alone. This indicates that $L_{r}$ and $L_{c}$ are complementary rather than redundant. Therefore, the ablation experiments comprehensively verify the design rationality of each module from multiple perspectives, providing solid experimental support for the overall architecture of the proposed model.

### 4.4. Impact of Hyperparameters

#### 4.4.1. The Impact of Different Masking Ratios

Table 8 and Table 9 illustrate the impact of different masking ratios γ on model performance for the ADS-B and Wi-Fi datasets, respectively. For the 30-class ADS-B task, the optimal masking ratio is γ = 0.4, achieving peak accuracy of 78.29%, 82.78%, 84.47%, and 88.00% across 10 to 25 labeled samples per class. This ratio balances the difficulty of local reconstruction with the integrity of global semantics, enabling robust feature extraction. For the 16-class Wi-Fi dataset, γ = 0.6 yields accuracies of 97.99%, 99.45%, 99.46%, and 99.72% under the same labeled sample sizes. It can be seen that excessively high or low masking ratios (e.g., 0.1 and 0.9) lead to significant performance degradation. When the masking ratio is too low, the model only needs to reconstruct local signal fragments, failing to compel the encoder to learn global dependency features of device fingerprints. Conversely, an excessively high ratio results in the over-masking of critical signal structures, causing both the reconstruction task and contrastive task to converge to suboptimal local solutions, thereby impairing the model’s ability to recover discriminative information effectively. Therefore, a moderate masking ratio (γ = 0.2 to 0.8) is considered appropriate, as it balances the difficulty of local reconstruction with the integrity of global semantics, enabling robust feature extraction and achieving higher accuracy.

#### 4.4.2. The Impact of Different Batch Sizes

**Figure 5:**
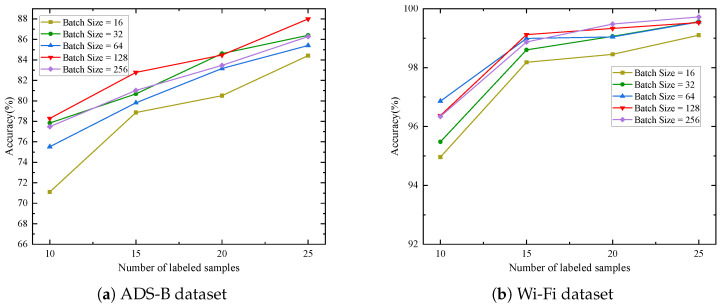
Recognition accuracy under different batch sizes.

Figure 5 illustrates the impact of different training batch sizes on model performance during the pre-training stage for the ADS-B and Wi-Fi datasets. Experimental results demonstrate that a batch size of 128 achieves the highest or near-highest recognition accuracy across varying numbers of labeled samples in both datasets. Specifically, on the ADS-B dataset, the batch size of 128 yields the highest accuracy for three cases (10, 15, and 25 labeled samples). On the Wi-Fi dataset, all batch sizes except for a batch size of 16 achieved high accuracy. Even with smaller batch sizes (such as 32), the model maintains high accuracy. These observations suggest that a batch size of 128 may provide relatively stable gradient estimates and sufficient update frequency. A moderate batch size can effectively balance gradient update stability and model generalization ability.

#### 4.4.3. The Impact of the Number of Unlabeled Samples

**Figure 6:**
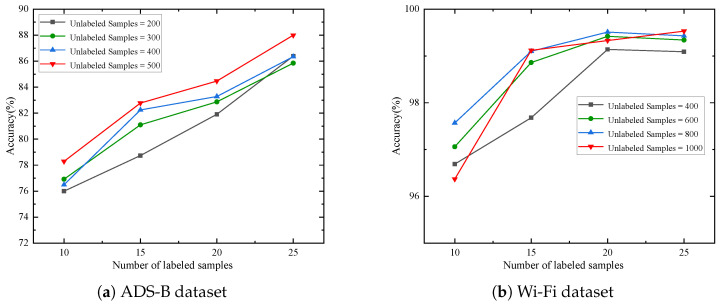
Recognition accuracy under different number of unlabeled samples.

The impact of the number of unlabeled samples on test accuracy is systematically evaluated for both ADS-B and Wi-Fi datasets, as shown in Figure 6. For the ADS-B dataset, raising unlabeled samples per class from 200 to 500 yields a clear upward trend in recognition accuracy. For example, with 10 labeled samples, accuracy improves from 76.00% to 78.29%. Similarly, for the Wi-Fi dataset, increasing unlabeled samples per class from 400 to 1000 generally boosts accuracy across most labeled sample counts. Notably, with 15 labeled samples, accuracy rises from 97.68% to 99.12%, and with 25 labeled samples, it increases from 99.09% to 99.53%. Although a minor fluctuation occurs for 10 labeled samples on the Wi-Fi dataset, the overall results confirm that self-supervised learning effectively extracts generalizable feature representations from unlabeled data, mitigating performance degradation due to labeled data scarcity.

### 4.5. Other Analyses

#### 4.5.1. The Impact of the Number of Projection Head Layers

To evaluate the impact of projection head layers on the proposed method, Table 10 and Table 11 present the recognition accuracy under different layer configurations for the ADS-B and Wi-Fi datasets. The results demonstrate that on ADS-B, a single FC layer achieves 88.00% accuracy with 25 labeled samples, while increasing the depth to two and three layers degrades performance by 3.31% and 4.78%, respectively, indicating overfitting risks. On the Wi-Fi dataset, although the two-layer head demonstrates better recognition performance, its advantage diminishes with increasing labeled samples. Crucially, the three-FC-layer configuration underperforms the single-layer head in all Wi-Fi scenarios. These findings confirm that excessively increasing projection head complexity hampers generalization.

#### 4.5.2. The Impact of Different Feature Encoders

Table 12 and Table 13 compare the recognition accuracy, number of parameters (Params), floating point operations (FLOPs), and training time per epoch for each class with 25 labeled samples of the proposed CSERNet with CVCNN [28], VGG [49], ResNet [50], and EfficientNet [51] when they are employed as feature encoders under varying numbers of labeled samples. The results show that on the ADS-B dataset, CSERNet significantly outperforms VGG, ResNet, and CVCNN models and performs better than EfficientNet when every class only has 10 and 25 samples. Specifically, with 10 labeled samples per class, CSERNet attains an accuracy of 78.29%, exhibiting improvements of 1.41%, 24.01%, 6.84%, and 0.55% over CVCNN, VGG, ResNet, and EfficientNet, respectively. On the Wi-Fi dataset, the performance of models like CVCNN and EfficientNet is slightly higher than that of CSERNet, but CSERNet remains highly competitive, exceeding 99% accuracy with 15 labeled samples per class. Crucially, CSERNet has the fewest parameters and the lowest FLOPs, but it still achieves the highest or a relatively high accuracy, demonstrating its robustness to model simplification. Additionally, its training time is shorter than that of CVCNN, ResNet, and EfficientNet, which validates the balance of CSERNet between high feature extraction capability and significantly lower resource requirements.

#### 4.5.3. The Impact of Self-Supervised Pre-Training

**Figure 7:**
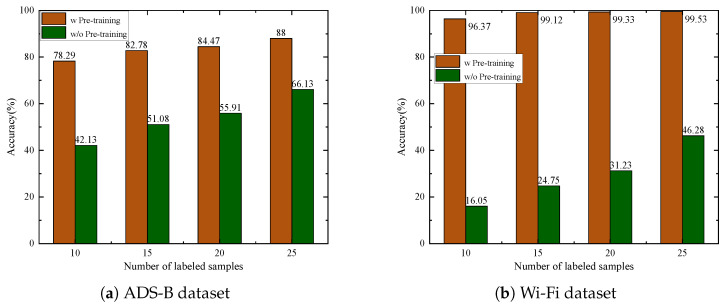
Recognition accuracy with/without pre-training.

To validate the effectiveness of the proposed self-supervised pre-training method, we compared it with a baseline approach that does not use pre-training. In the baseline, both the feature encoder and classifier parameters are randomly initialized and then fine-tuned using 10, 15, 20, or 25 labeled samples. The experimental results shown in Figure 7 on both datasets demonstrate that the proposed self-supervised pre-training method consistently outperforms the baseline across various scenarios. Specifically, on the ADS-B dataset, with 10 labeled samples, the pre-trained model achieves an accuracy of 78.29%, which is 36.16% higher than the baseline without pre-training. On the Wi-Fi dataset, with 10 labeled samples, the pre-trained model achieves an accuracy of 96.37%, while the baseline without pre-training is only 16.05%. In scenarios with 15, 20, and 25 labeled samples, the proposed method continues to maintain robust performance. These results clearly indicate that the proposed method has significant advantages in scenarios with limited labeled samples. This validates its ability to maintain high performance with fewer labeled samples while effectively leveraging unlabeled data to maximize its potential.

#### 4.5.4. The Impact of Masking Mode

**Figure 8:**
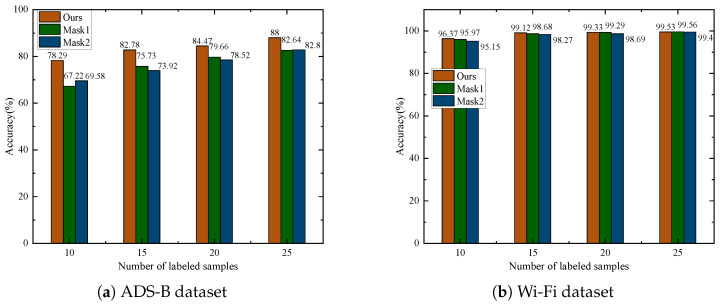
Impact of masking mode.

This experiment aims to validate the effectiveness of the proposed complementary masking mode. Two baseline methods are designed: Mask1, which applies random masking with identical masking ratios to both augmented signals, and Mask2, which employs random masking with masking ratios summing to 1 for the two augmented signals. The experimental results shown in Figure 8 demonstrate that the proposed complementary masking strategy achieves better performance under the condition of having very few labeled samples per class (e.g., 10 or 15). On the ADS-B dataset, when the number of labeled samples per class is 25, the proposed method achieves an accuracy of 88.00%, which is 5.36% and 5.2% higher than the other two methods, respectively. With only 10 labeled samples, the performance gap becomes more pronounced, where the proposed method achieves improvements of 11.07% and 8.71% over Mask1 and Mask2. On the Wi-Fi dataset, with only 10 labeled samples per class, the proposed method achieves 0.4% and 1.22% higher accuracy than Mask1 and Mask2, respectively. The experimental results indicate that the complementary masking mechanism enhances the robustness of feature learning, thus making it more adaptable to scenarios with scarce labeled data.

#### 4.5.5. The Impact of Encoder–Decoder Symmetry

To validate the effectiveness of the proposed asymmetric encoder–decoder architecture, its performance is compared against a symmetric architecture. Results based on the ADS-B and Wi-Fi datasets are presented in Table 14 and Table 15, clearly demonstrating the advantage of the asymmetric design. On the ADS-B dataset, the asymmetric architecture consistently outperforms the symmetric counterpart across all labeled sample scenarios, with a performance improvement of 6.25% to 8.96%. Similarly, on the Wi-Fi dataset, the asymmetric architecture maintains performance advantages, achieving a 3.57% advantage with 10 samples per class. While the performance difference diminished as the number of labeled samples increased to 25, the asymmetric design still achieved marginally higher accuracy. The asymmetric architecture effectively reduces model complexity and computational overhead by employing a powerful encoder to extract rich features while utilizing a simple decoder focused solely on reconstruction. This design mitigates overfitting of the complex decoder to the reconstruction task, facilitating the learning of more discriminative and generalizable feature representations by the encoders.

#### 4.5.6. The Impact of Signal Length

Table 16 presents the recognition performance of the model on the ADS-B dataset under three different input signal lengths: *L* = 3750, *L* = 4000, and *L* = 4250. As can be seen from the table, when there are 10 labeled samples per class, the model achieves the highest recognition accuracy under the condition of *L* = 4000. When there are 15, 20, and 25 labeled samples per class, the model achieves the highest recognition accuracy under the condition of *L* = 4250. Under the four few-shot conditions, the model is able to maintain a high level of recognition accuracy under different signal lengths, and the maximum fluctuation in the model’s recognition performance across different signal lengths is approximately 3%. This demonstrates that the CAML-SEI model exhibits certain robustness to changes in signal length. Its self-supervised feature extraction mechanism does not strictly depend on a fixed-length signal segment. The model is able to learn effective RFF features from signals of different lengths.

## 5. Conclusions

Aiming at the challenge of scarce labeled samples in SEI tasks under non-cooperative communication scenarios, this paper proposes a self-supervised learning method based on contrastive asymmetric masked learning. The method innovatively integrates dual optimization mechanisms of masked signal reconstruction and contrastive learning. Through complementary masking augmentation strategies, it preserves critical fingerprint information in signals while utilizing masked regions to guide robust feature learning. Furthermore, a channel squeeze-and-excitation residual network combined with an asymmetric encoder–decoder architecture is designed to enhance key feature representation while reducing model complexity, significantly improving feature extraction capability. Experimental results demonstrate that the proposed CAML-SEI method outperforms other comparative methods across multiple extreme labeled conditions (only 10, 15, 20, 25 samples per category) on both the 30-class ADS-B dataset and 16-class Wi-Fi dataset. This conclusively verifies its superiority in learning robust and generalizable fingerprint characteristics for RFFs, providing a reliable technical solution for addressing RFF feature extraction challenges in label-scarce scenarios.

The proposed CAML-SEI method offers several advantages, and our experiments have validated its robustness under moderate SNR conditions. However, performance may not be optimal under low SNR conditions, for example, at SNR levels below 0 dB. Therefore, future work will integrate a signal denoising module to enhance robustness for low SNR conditions. Simultaneously, considering the issue of signal interference in non-cooperative scenarios, we will incorporate interference detection as an auxiliary pre-training task in the self-supervised framework to improve model performance. Moreover, regarding future research directions, we will focus on the study of cross-domain SEI, specifically investigating whether pre-trained models can be adapted to SEI recognition tasks where signal distributions may change over time. To address the cross-domain SEI problem, we plan to introduce domain adaptation techniques in future work. Additionally, we will employ model compression strategies such as model pruning, quantization, and knowledge distillation to further reduce model size, enabling edge deployment. Furthermore, for ultra-large-scale device identification scenarios, we will explore combining memory bank mechanisms with adaptive hard sample mining to further improve the quality of negative samples and training scalability. 

## Figures and Tables

**Table 1:** Network structure of CAML-SEI method.

Network Structure	Layer	Number
Encoder	Conv1d+BatchNorm+ReLU+Maxpool	1
	CSERBlock	7
	Flatten+FC	1
Projection Head	FC	1
Decoder	Conv1d	1
Classifier	Dropout+FC	1

**Table 2:** Recognition accuracy of different methods on the ADS-B dataset.

Method	Number of Labeled Samples			
	10	15	20	25
AE	44.89% ± 5.17%	53.50% ± 4.41%	60.04% ± 3.78%	67.33% ± 5.63%
DCGAN	46.95% ± 2.23%	54.48% ± 3.16%	58.53% ± 2.59%	64.07% ± 1.88%
MAE	59.02% ± 2.30%	66.87% ± 2.75%	71.55% ± 2.34%	77.24% ± 1.52%
AMAE	74.04% ± 1.75%	78.11% ± 1.15%	81.28% ± 1.28%	85.26% ± 1.10%
BYOL	41.38% ± 3.11%	50.15% ± 2.01%	57.60% ± 3.15%	67.80% ± 2.00%
SimCLR	63.59% ± 2.79%	72.17% ± 2.69%	75.59% ± 2.50%	82.37% ± 1.96%
SA2SEI	43.39% ± 1.71%	57.23% ± 1.01%	68.17% ± 1.90%	77.38% ± 1.13%
CAML-SEI	78.29% ± 1.99%	82.78% ± 2.86%	84.47% ± 2.14%	88.00% ± 1.72%

**Table 3:** Recognition accuracy of different methods on the Wi-Fi dataset.

Method	Number of Labeled Samples			
	10	15	20	25
AE	25.60% ± 9.77%	40.82% ± 8.11%	47.12% ± 11.56%	52.24% ± 6.61%
DCGAN	46.71% ± 4.85%	50.23% ± 2.56%	56.58% ± 1.84%	60.18% ± 2.35%
MAE	92.14% ± 3.76%	97.57% ± 1.09%	98.51% ± 0.90%	98.87% ± 0.89%
AMAE	75.61% ± 10.93%	86.74% ± 2.36%	90.89% ± 1.86%	91.89% ± 1.26%
BYOL	13.66% ± 3.46%	21.99% ± 2.33%	24.35% ± 2.74%	40.99% ± 2.70%
SimCLR	85.92% ± 8.82%	97.57% ± 0.70%	97.76% ± 0.79%	99.04% ± 0.57%
SA2SEI	57.68% ± 1.96%	66.26% ± 2.31%	74.38% ± 1.91%	75.35% ± 4.19%
CAML-SEI	96.37% ± 2.20%	99.12% ± 0.44%	99.33% ± 0.50%	99.53% ± 0.35%

**Table 4:** F1-score and recall of the CAML-SEI method on the ADS-B dataset.

Dataset	Number of Labeled Samples			
	10	15	20	25
F1-score	78.14%	82.70%	84.40%	87.99%
Recall	78.29%	82.78%	84.48%	88.00%

**Table 5:** F1-score and recall of the CAML-SEI method on the Wi-Fi dataset.

Dataset	Number of Labeled Samples			
	10	15	20	25
F1-score	96.32%	99.11%	99.33%	99.53%
Recall	96.37%	99.12%	99.33%	99.53%

**Table 6:** Ablation experiment on the ADS-B dataset.

Method	Number of Labeled Samples			
	10	15	20	25
Ours	78.29%	82.78%	84.47%	88.00%
Ours w/o CSE	67.30%	75.02%	78.71%	83.14%
Ours w/o Proh	77.53%	81.17%	84.39%	86.71%
Ours w/o $L_{r}$	72.65%	77.33%	80.61%	84.06%
Ours w/o $L_{c}$	64.20%	72.28%	76.14%	79.24%
only $L_{r}$	62.55%	72.41%	76.29%	79.65%
only $L_{c}$	72.61%	74.53%	78.03%	80.13%

**Table 7:** Ablation experiment on the Wi-Fi dataset.

Method	Number of Labeled Samples			
	10	15	20	25
Ours	96.37%	99.12%	99.33%	99.53%
Ours w/o CSE	91.41%	96.24%	97.33%	97.91%
Ours w/o Proh	96.26%	98.36%	98.94%	99.49%
Ours w/o $L_{r}$	92.62%	98.22%	98.88%	99.42%
Ours w/o $L_{c}$	59.25%	72.03%	79.31%	82.31%
only $L_{r}$	65.65%	79.89%	87.15%	92.17%
only $L_{c}$	90.24%	94.97%	96.36%	97.93%

**Table 8:** Recognition accuracy with different masking ratios on the ADS-B dataset.

γ	Number of Labeled Samples			
	10	15	20	25
0.1	47.04%	57.98%	69.12%	76.81%
0.2	77.83%	81.38%	83.62%	85.93%
0.3	77.24%	81.86%	84.68%	87.09%
0.4	78.29%	82.78%	84.47%	88.00%
0.5	76.46%	80.54%	82.87%	84.27%
0.6	77.57%	81.38%	84.32%	87.36%
0.7	77.51%	80.02%	82.98%	85.98%
0.8	73.81%	78.82%	81.55%	84.39%
0.9	44.72%	59.31%	66.92%	76.03%

**Table 9:** Recognition accuracy with different masking ratios on the Wi-Fi dataset.

γ	Number of Labeled Samples			
	10	15	20	25
0.1	96.21%	99.12%	99.10%	99.43%
0.2	96.60%	99.18%	99.63%	99.75%
0.3	96.18%	98.95%	99.21%	99.49%
0.4	96.37%	99.12%	99.33%	99.53%
0.5	96.98%	98.61%	99.40%	99.56%
0.6	97.99%	99.45%	99.46%	99.72%
0.7	97.63%	99.06%	99.32%	99.58%
0.8	95.93%	99.38%	99.70%	99.71%
0.9	94.66%	98.92%	99.02%	99.63%

**Table 10:** Impact of projection head layers on the ADS-B dataset.

FC Layers	Number of Labeled Samples			
	10	15	20	25
1	78.29%	82.78%	84.47%	88.00%
2	73.91%	78.62%	82.71%	84.69%
3	67.79%	75.72%	79.37%	83.22%

**Table 11:** Impact of projection head layers on the Wi-Fi dataset.

FC Layers	Number of Labeled Samples			
	10	15	20	25
1	96.37%	99.12%	99.33%	99.53%
2	98.70%	99.49%	99.41%	99.71%
3	95.49%	98.50%	99.34%	99.42%

**Table 12:** Impact of different encoders on the ADS-B dataset.

Model	Number of Labeled Samples				FLOPs (M)	Params (K)	Time (s)
	10	15	20	25			
CVCNN	76.88%	81.52%	83.83%	87.28%	266.62	791.42	0.49
VGG	54.28%	63.81%	68.28%	72.04%	172.60	3990.81	0.21
ResNet	71.45%	80.14%	84.54%	87.36%	1371.62	3844.35	0.60
EfficientNet	77.74%	83.41%	85.98%	87.33%	498.75	3606.81	0.74
CSERNet	78.29%	82.78%	84.47%	88.00%	168.61	751.97	0.24

**Table 13:** Impact of different encoders on the Wi-Fi dataset.

Model	Number of Labeled Samples				FLOPs (M)	Params (K)	Time (s)
	10	15	20	25			
CVCNN	97.90%	99.25%	99.38%	99.54%	266.62	791.42	0.27
VGG	95.61%	97.39%	97.55%	98.67%	172.60	3990.81	0.12
ResNet	97.30%	98.34%	99.10%	99.40%	1371.62	3844.35	0.33
EfficientNet	97.34%	98.88%	99.30%	99.59%	498.75	3606.81	0.41
CSERNet	96.37%	99.12%	99.33%	99.53%	168.61	751.97	0.13

**Table 14:** Impact of encoder–decoder symmetry on the ADS-B dataset.

Method	Number of Labeled Samples			
	10	15	20	25
Asymmetric	78.29%	82.78%	84.47%	88.00%
Symmetric	72.04%	74.69%	76.97%	79.04%

**Table 15:** Impact of encoder–decoder symmetry on the Wi-Fi dataset.

Method	Number of Labeled Samples			
	10	15	20	25
Asymmetric	96.37%	99.12%	99.33%	99.53%
Symmetric	92.80%	98.79%	98.40%	99.51%

**Table 16:** Impact of signal length.

Method	Number of Labeled Samples			
	10	15	20	25
L=3750	75.21%	81.48%	86.20%	89.37%
L=4000	78.29%	82.78%	84.47%	88.00%
L=4250	77.28%	83.81%	87.59%	90.34%

## Data Availability

The raw data supporting the conclusions of this article will be made available by the authors on request.
